# Recent developments in human cell models in mental disorders

**DOI:** 10.1016/j.nsa.2024.103939

**Published:** 2024-01-11

**Authors:** Sarah Kittel-Schneider

**Affiliations:** Department of Psychiatry and Neurobehavioural Science, University College Cork, Acute Adult Mental Health Unit, Cork University Hospital, T12DC4A, Wilton, Cork, Ireland

The increasing neurobiological understanding of mental health disorders means that there are sufficient promising therapeutic targets and mechanisms known from which to design new therapies. However, the major challenge is their validation and translation into effective medicine that works in patients. Although animal studies are an important component of early-stage mechanistic research, they perform poorly in predicting clinical efficacy, with 90% of drug candidates selected after preclinical animal testing failing to translate to the human condition (Zhu, 2020). This deficit in the drug development value chain was recently recognized in the US with the dropping of the legal requirement for animal testing by the FDA (Wadman, 2023). Recent human induced pluripotent stem cell (hiPSC) research has allowed for more human-centric models to be utilized in the field. HiPSC-derived cell models, 2D monoculture, complex 2D co-cultures, and 3D models such as organoids, can be highly attractive for preclinical screening, bridging the gap between *in-vitro* and *in-vivo* studies and reducing animal testing. However, as the hiPSC research of neurodevelopmental and mental disorders is still in its infancy, there are still challenges to overcome and first results need to be replicated. Recently, within the iPSCs Platform for Neuropsychiatry ECNP Network , we have summarised recommendations for best practice in hiPSC studies in neuropsychiatry. In short, we recommend a very careful donor/phenotype selection, including sex as an important biological variable, increase biological and not technical replicates and careful selection of controls. A very useful and sensible approach to power calculation of hiPSC studies has been recently described by Brunner et al. (2023). If possible, then the use of isogenic controls might also reduce the needed numbers of biological replicates, if not, then sex matched family members might be potentially better than random healthy control donors. In pharmacological studies, cellular read-outs associated with potential drug response need to be defined first as well as time- and concentration (dose-)response curves need to be evaluated. Also, the time point in the maturation of the cells need to be considered for high throughput drug screening (Polit et al., 2023). Besides developing models for the more successful and accelerated development of novel treatments, hiPSC studies can also enlighten further molecular and cellular disease-associated mechanisms and mode of actions of effective drugs. The latter is also important to understand response and non-response mechanisms towards the development of predictive biomarkers. Furthermore, hiPSC models can lead to a better understanding of the contribution of genetic risk variants to a disorder in the human context in contrast to genetic animal models. As an example, variants in the Cadherin-13 (*CDH-13*) gene have been associated with as well attention-deficit/hyperactivity disorder (ADHD) and common comorbid disorders (Rivero et al., 2015), cognitive function and personality traits in ADHD (Ziegler et al., 2021). In a human cell model the involvement in GABAergic modulation as been shown (Mossink et al., 2022). The most recent results from Vitale et al. confirm the influence of allelic variants of *CDH-13*, a calcium-dependent cell adhesion molecule specifically expressed in GABAergic synapses, on the homeostasis of excitatory/inhibitory (E/I) networks and strengthened the hypothesis of glutamatergic/GABAergic pathomechanisms playing a role in neurodevelopmental and mental disorders (Vitale et al.).

Besides a dysregulated E/I balance, there are other pathomechanisms suggested to be involved in the etiology of ADHD. Grünblatt et al. have previously suggested that alterations in Wnt signalling may be involved in childhood ADHD (Yde Ohki et al., 2020). Previous findings in SH-SY5Y cells suggested that methylphenidate may exert its effects by influencing Wnt signalling (Grunblatt et al., 2018). Nevertheless, as the cell lines utilized were neuroblastoma cell lines, it is imperative to validate these findings using neural cells derived from hiPSC obtained from actual individuals with ADHD, as these cells more closely resemble brain cells in human patients. In their latest work on ADHD patients, Walter et al. have demonstrated that there are certainly alterations to Wnt signaling compared with cells from neurotypical children. These abnormalities are particularly evident during the neural stem cell (NSC) development phase. Interestingly, the changes in protein levels and Wnt-activity were specifically linked to the genetic susceptibility to ADHD and comorbid disorders, rather than unrelated conditions such as baldness. Furthermore, the examination of the polygenic scores specific to the Wnt pathway revealed a correlation between clinical and cellular parameters, suggesting a possible link between ADHD and modified Wnt pathway (Walter et al.).

A common comorbidity of ADHD is major depressive disorder (MDD) (Riglin et al., 2021; Shapero et al., 2021). This is, besides anxiety disorders, the most common mental health condition worldwide. As only about 30% of individuals respond sufficiently to the first antidepressant treatment and about 10–15 % do not have a sufficient response to multiple treatment trials and are considered affected by treatment-resistant depression (TRD) or difficult-to-treat depression, there is a great unmet medical need for more precise and also novel treatment approaches.

In patients with MDD-TRD, ketamine was shown to produce rapid antidepressant effects (Zarate et al., 2006) and (Es-)Ketamine is now a recognized treatment for MDD-TRD. However, the exact mode of action is still unclear.

In a previous study Cavalleri et al. (2018) showed that exposure to ketamine enhances structural neural plasticity of human dopaminergic (DA) neurons differentiated from hiPSC via AMPA receptor and BDNF-mTOR signaling. In their recent work Cavalleri et al. extended their study and showed first evidence that 1 hr exposure to ketamine or its metabolite (2R,6R)-hydroxynorketamine reverted the impaired dendritic arborization produced by cortisol at doses 10–100-fold lower than those effective in DA neurons not exposed to cortisol. Interestingly these doses are fully within the range of exposure in patients (Cavalleri et al.).

In summary, hiPSC models have not only the potential to enhance the knowledge about molecular and cellular processes of neurodevelopmental and mental disorders, but also reveal potential new drug targets and elucidate cellular response and non-response mechanisms. In the near future, we will be able to generate high-throughput drug screening tools, to reduce, refine and replace animal models and to achieve a higher success in drug development towards a more individualised treatment approach.

## Declaration of competing interest

SKS has received speaker's honoraria from Medice Arzneimittel Puetter GmbH & CoKG, Takeda and Janssen in the past 3 years.
